# Supplementary figures and images for: Identification of Austwickia chelonae as cause of cutaneous granuloma in endangered crocodile lizards using metataxonomics
Source: PeerJ. 2019 Mar 13;7:e6574. doi: 10.7717/peerj.6574 (PMC6420803; doi:10.7717/peerj.6574)

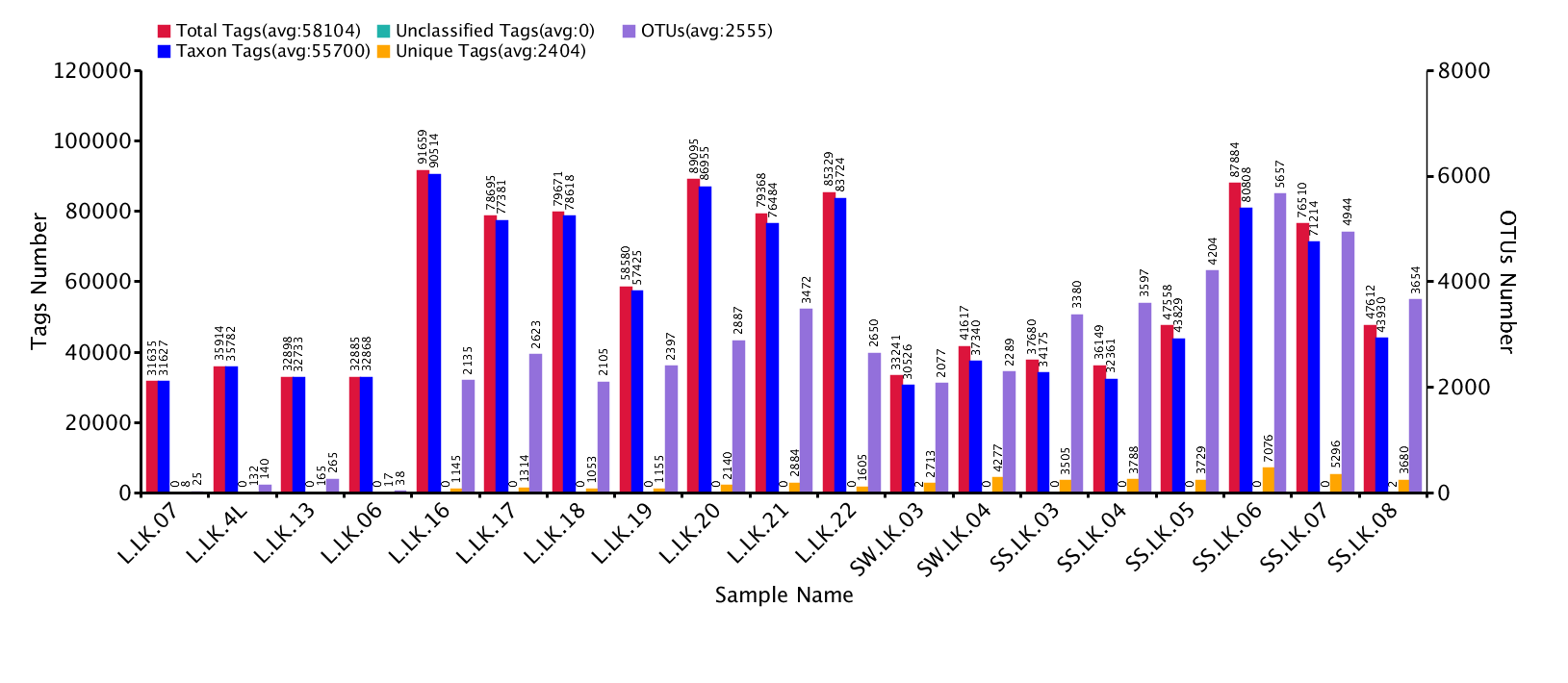

Supplement: Figure S1 — Total tags (red, indicates effective tags) were sequences without low-quality sequences and chimeras and were used for annotation and other analyses. Taxon tags (blue) represent the sequences that could be clustered into OTUs and annotated. Unclassified tags (green) refer to tags without annotated information. Unique tags (orange) refer to tags that occurred only once and could not be clustered into the number of OTU tags. [file peerj-07-6574-s002.png]

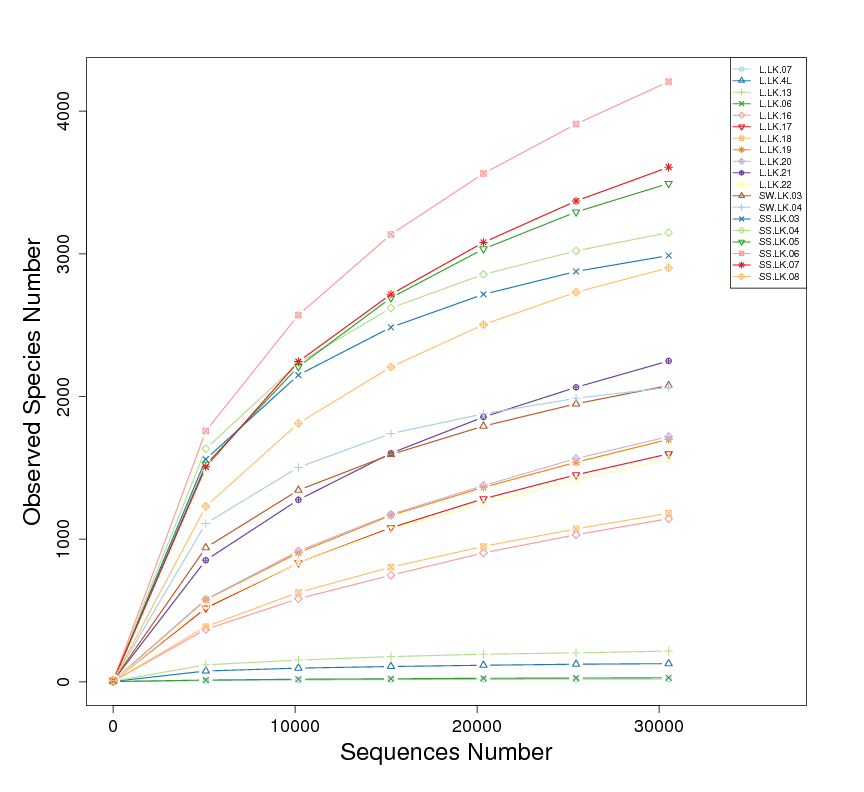

Supplement: Figure S2 [file peerj-07-6574-s003.png]

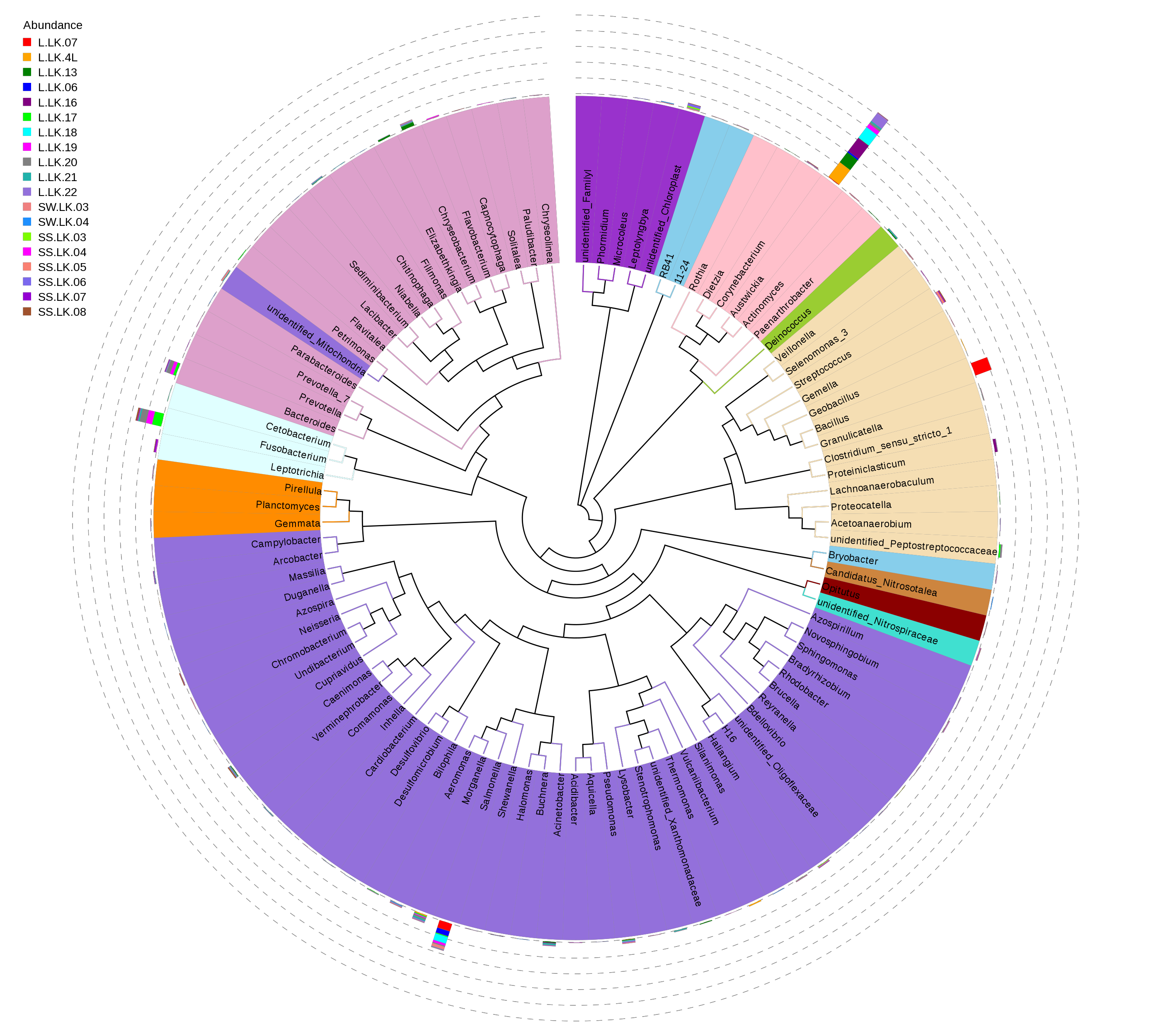

Supplement: Figure S3 — The branches were colored by phyla. The bars out of the branches represent relative abundances and colored by samples. [file peerj-07-6574-s004.png]
